# Temperature effect on tert-butyl alcohol (TBA) biodegradation kinetics in hyporheic zone soils

**DOI:** 10.1186/1475-925X-6-34

**Published:** 2007-09-19

**Authors:** Mark H Greenwood, Ronald C Sims, Joan E McLean, William J Doucette

**Affiliations:** 1 Division of Environmental Engineering, Utah State University, Logan, Utah 84321, USA; 2 Utah Water Research Laboratory, Utah State University, Logan, Utah 84321, USA; 3 Department of Biological and Irrigation Engineering, Logan, Utah 84321, USA

## Abstract

**Background:**

Remediation of tert-butyl alcohol (TBA) in subsurface waters should be taken into consideration at reformulated gasoline contaminated sites since it is a biodegradation intermediate of methyl tert-butyl ether (MTBE), ethyl tert-butyl ether (ETBE), and tert-butyl formate (TBF). The effect of temperature on TBA biodegradation has not been not been published in the literature.

**Methods:**

Biodegradation of [U ^14^C] TBA was determined using hyporheic zone soil microcosms.

**Results:**

First order mineralization rate constants of TBA at 5°C, 15°C and 25°C were 7.84 ± 0.14 × 10^-3^, 9.07 ± 0.09 × 10^-3^, and 15.3 ± 0.3 × 10^-3 ^days-1, respectively (or 2.86 ± 0.05, 3.31 ± 0.03, 5.60 ± 0.14 years^-1^, respectively). Temperature had a statistically significant effect on the mineralization rates and was modelled using the Arrhenius equation with frequency factor (A) and activation energy (Ea) of 154 day^-1 ^and 23,006 mol/J, respectively.

**Conclusion:**

Results of this study are the first to determine mineralization rates of TBA for different temperatures. The kinetic rates determined in this study can be used in groundwater fate and transport modelling of TBA at the Ronan, MT site and provide an estimate for TBA removal at other similar shallow aquifer sites and hyporheic zones as a function of seasonal change in temperature.

## Background

The presence of methyl tert-butyl ether (MTBE) and the biodegradation intermediate tert-butyl alcohol (TBA) in shallow aquifer systems affected by seasonal low temperature groundwater (~5°C) have been widely reported [e.g. [1,2]]. In the past, the use of monitored natural attenuation (MNA) as a remediation alternative at MTBE and TBA contaminated sites in low temperature climates has had questionable utility because of the low rate of biodegradation anticipated to occur at low temperatures.

Mesophilic microbial communities show optimum growth and biodegradation of substrates from 20°C to 40°C and become ineffective at 5°C [3,4]. Subsurface contaminant remediation with winter temperatures below 5°C would become temporarily depressed through part of the year since these systems are predominated by mesophilic microbial communities. However, MNA may remain an effective remediation year round if the microbial community is psychrotolerant, which is characteristic of having optimum temperature ranges from 15°C to 30°C and becoming ineffective at 0°C [4].

Multiple studies have reported the bioremediation of groundwater contaminants including MTBE, aromatic hydrocarbons, and alkanes at or below 5°C with significant removal [5-8], which demonstrates the applicability of MNA at contaminated groundwater sites in lower temperature regions. Significant biodegradation of MTBE from 4°C up to 34°C was observed by Bradley and Landmeyer [5] in soils from a MTBE contaminated site in Ronan, Montana. The effect of temperature on TBA biodegradation, the primary degradation intermediate of MTBE, has not been published in the literature. The presence of TBA in shallow aquifers at low temperatures may be the rate limiting step for remediating a MTBE contaminated site using MNA. TBA can be stoichiometrically formed not only from MTBE but also from ethyl tert-butyl ether (ETBE) and tert-butyl formate (TBF), which are now used as fuel additives in place of MTBE [9]. Also, TBA has been found to be present in gasoline up to 11% of the volume of MTBE as an impurity [10]. Therefore, the presence of TBA, a potential human carcinogen [11] and one of the most mobile gasoline LUFT contaminants [12], in the environment could soon be more pertinent than that of MTBE.

The purpose of this study was to observe the effect of temperature on TBA biodegradation kinetics in soils within the hyporheic zone of the shallow MTBE and TBA contaminated aquifer in Ronan, MT. The hyporheic zone is defined as the region of interaction between groundwater (low dissolved oxygen (DO) and high in nutrients) and surface water (high DO and low in nutrients). Hyporheic zones have been observed to exhibited increase in the rate of biodegradation of dissolved organic carbon (DOC) that was not otherwise degraded in surface and subsurface waters [13-15]. The authors attributed the increased biodegradation rate to the interaction of the groundwater and surface water where exchanges of nutrients, oxygen, and organic matter stimulated hyporheic zone microbe activity. This study examined the effect of temperature range from 5 to 25°C on the degradation rate of TBA in aerobic microcosms containing soil collected from the Ronan hyporheic zone.

## Methods

### Chemicals

TBA (>99.9% HPLC grade) was purchased from Sigma Aldrich Chemical Co., Bellefont PA and was used as received. Uniformly labelled [U ^14^C] TBA (> 98% purity; 4.0 mCui mmol^-1^) was obtained from Nuclear Research Products, Du Pont, Boston, MA.

### Study site

**Figure 1:**
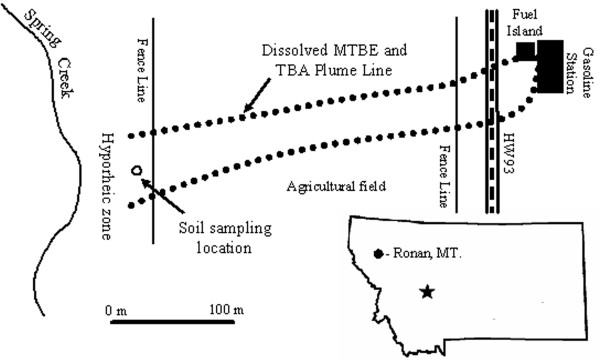
Ronan, MT MTBE plume site and hyporheic zone.

The MTBE contaminated site in Ronan, Montana (located 170 kilometers north of Missoula, Montana) was created by a leaking underground fuel tank (LUFT) that released approximately 22,000 litters of gasoline in 1994 [16]. The spill created an MTBE free product plume that moved west under Highway 93 and a dissolved plume that travelled under an agricultural field towards Spring Creek located approximately 460 meters west of the spill site (Figure 1). The contaminated aquifer is up to 5 meters below ground surface (bgs) near the gasoline station and at surface discharge (zero m bgs) in the Spring Creek hyporheic zone located between the creek and the agricultural field fence line [16]. Groundwater concentrations of MTBE and TBA in the hyporheic zone were 1000 μg/L and 500 μg/L, respectively, in 2002. The maximum dissolved concentrations of MTBE and TBA reported at the site were 7000 μg/L and 1000 μg/L, respectively.

All contaminated groundwater at the site enters Spring Creek via the hyporheic zone, which creates a seasonal wetland between Spring Creek and fence line. Groundwater discharging into Spring Creek, after passing through the hyporheic zone, contains only 300 μg/L MTBE and non-detectable (< 5 μg/L) concentration of TBA. However, the growth of the TBA plume may result in increased flux of TBA into Spring Creek [16].

### Soil/aquifer materials

A soil sample was collected 30 cm bgs in the hyporheic zone of the Ronan, MT site along the centreline of the MTBE plume (Figure 1). The soil sample was taken approximately 20 cm bgs and stored in sterilized glass containers at 4°C in the dark. Soil characteristics are pH of 7.7, percent organic carbon of 6.80%, and percent sand, silt, and clay of 27%, 51%, and 22%, respectively with nutrient and chemical concentrations of 46 mg/kg phosphorous, 144 mg/kg potassium, 6.11 mg/kg nitrate, 3.18 mg/kg zinc, 200 mg/kg total iron, 3.21 mg/kg copper, 44.6 mg/kg manganese, and 25.4 mg/kg sulfate.

### Microcosm experiment

**Figure 2:**
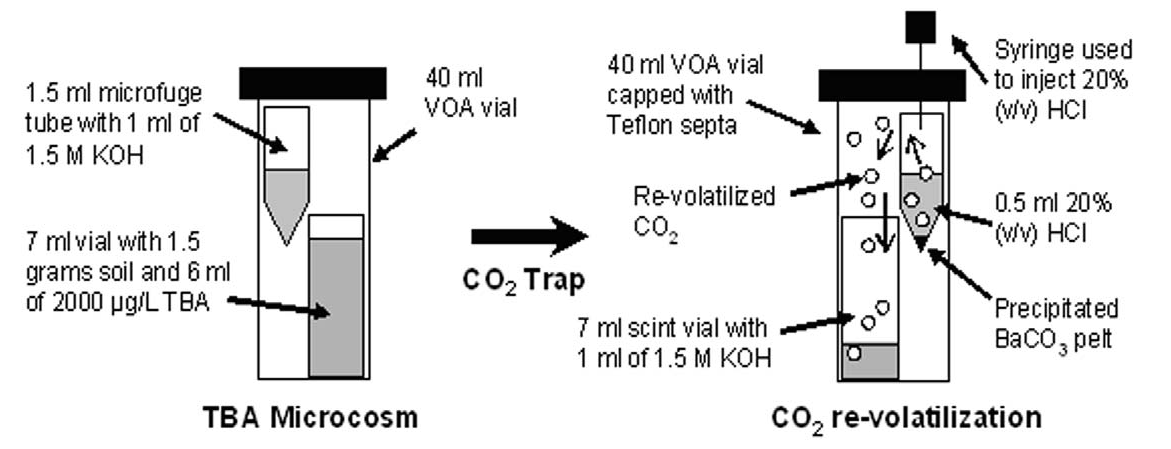
Experimental microcosm and CO_2 _re-volatilization setups.

Each microcosm consisted of a 40-ml volatile organic air-tight (VOA) vial with one 7-ml vial and a 1.5-ml microfuge tube (Figure 2). The 7-ml vial contained 1.5 grams of soil with 5 ml of 2000 μg/L TBA solution and 0.1 μCi [U ^14^C] TBA. The 1.5 ml centrifuge tube contained 1 ml of 1.5 M potassium hydroxide (KOH) solution used to trap carbon dioxide (CO_2_) (Figure 2).

After collection of CO_2 _in KOH, 0.5 ml of 1.5 M barium chloride (BaCl) was added to each trap to precipitate ^14^CO_2 _to form insoluble barium carbonate. After centrifugation at 13 gs for 10 minutes, supernatant containing [U ^14^C] TBA was pored off leaving the barium carbonate pelt at the bottom. Each pelt was re-volatilized with 0.25 ml of 20% (v/v) hydrochloric acid in a 40-ml VOA vial containing a 7-ml glass vial with 1 ml of 0.5 M KOH solution to capture ^14^CO_2 _(Figure 2). After equilibration on a shaker table for 24 hrs, 6 mls of scintillation cocktail was added to the 7 ml vial and analyzed by liquid scintillation counting. It is important to note that, using this indirect way, only the carbon of mineralized TBA (^14^CO_2_) was measured and not [U ^14^C] TBA.

Microcosms were incubated at 5, 15, and 25°C in the dark and CO_2 _traps were replaced every 4–8 days for 220 days (with exception of sampling times 200 and 220, where traps where replaced after 20 days). The continual replacement of the CO_2 _traps every 4–8 days provided exposure of the system to atmospheric oxygen to maintain oxic conditions. Poisoned controls, non-poisoned controls, soil slurry blanks (soil slurry with no TBA), and water spikes (water with TBA only) were used to confirm biological degradation of TBA and to prevent false positives. The soil was poisoned with mercuric chloride (HgCl_2_) at 1000 mg/kg soil and autoclaved twice for one hour each. Final concentrations of [U ^14^C] TBA in the microcosms where measured by scintillation counting of 1 ml of solution in 6 ml of scintillation cocktail.

## Results

Total mineralization of TBA in non-poisoned soil microcosms at 5°C, 15°C, and 25°C after 220 days was 54.0%, 64.4%, and 69.8%, respectively (initial concentration of 2000 μg/L). All control samples (including poisoned microcosms) had zero readings of ^14^CO_2_. The non-poisoned microcosm soil slurry was measured on day 220 with no detectable scintillation counts. Therefore, 46.0%, 35.6% and 30.2% of the [U 14C] TBA at 5°C, 15°C, and 25°C, respectively, were not recovered as ^14^CO_2_.

Since TBA is miscible in water with a Henry's constant of 4.9 × 10^-4 ^atm-m3/mol (at 25°C), it is assumed that the compound would remain in solution and only a small amount would partition into the headspace. Assuming that all of the headspace in the microcosm (volume of 30 ml) was replaced with ambient air (void of TBA) every time the CO_2 _traps were replaced and the headspace (not including the CO_2 _trap solution) had reached equilibrium with the slurry, less than 0.01% of the TBA would have been removed from the microcosms at an incubation temperature of 25°C.

Therefore, it was assumed that TBA in the slurry reached equilibrium with the CO_2 _trap along with the headspace in the microcosm. This was observed in an experiment where the supernatant from poisoned microcosm (no biological activity) CO_2 _traps were counted after 4, 11, and 18 days of incubation under identical conditions in the 5°C, 15°C, and 25°C microcosms studies. With an initial concentration of 2000 μg/L in the soil slurry, the cumulative percent removal of TBA via partitioning (i.e. percent TBA detected in the CO_2 _trap) in the 5°C, 15°C, and 25°C poisoned microcosms was 18.5%, 23.5%, and 29.6%, respectively. Therefore, the determination of the first order mineralization rate was corrected for the removal of TBA via partitioning into the CO_2 _trap.

### Theory

The change in concentration of TBA over time in the microcosm was caused by biodegradation and partitioning of TBA into the CO_2 _trap. The rate of change equation for this system is

dCdt=km⋅C+rp⋅C
 MathType@MTEF@5@5@+=feaafiart1ev1aaatCvAUfKttLearuWrP9MDH5MBPbIqV92AaeXatLxBI9gBaebbnrfifHhDYfgasaacH8akY=wiFfYdH8Gipec8Eeeu0xXdbba9frFj0=OqFfea0dXdd9vqai=hGuQ8kuc9pgc9s8qqaq=dirpe0xb9q8qiLsFr0=vr0=vr0dc8meaabaqaciaacaGaaeqabaqabeGadaaakeaadaWcaaqaaiabdsgaKjabdoeadbqaaiabdsgaKjabdsha0baacqGH9aqpcqWGRbWAdaWgaaWcbaGaemyBa0gabeaakiabgwSixlabdoeadjabgUcaRiabdkhaYnaaBaaaleaacqWGWbaCaeqaaOGaeyyXICTaem4qameaaa@407C@

where C is the concentration of TBA (μg/L) in the water soil slurry, dC/dt is the rate of change of the concentration of TBA over time, and k_m _(day^-1^) and r_p _(day^-1^) are the first order rate constants of mineralization and partitioning of TBA, respectively. This model assumes that TBA mineralization follows first order kinetics and that TBA loss via partitioning can be modelled by a first order constant (i.e. the actual rate of removal of TBA via partitioning will change over time and can only be averaged over the incubation time). Since the partitioning rate constant is a pseudo first order rate constant, it is represented by r_p _and not k_p _(first order rate of partitioning). Integration and manipulation of equation 1 provides the change in total mass of TBA with respect to time

Ms=Mso⋅e−(km+rp)⋅t
 MathType@MTEF@5@5@+=feaafiart1ev1aaatCvAUfKttLearuWrP9MDH5MBPbIqV92AaeXatLxBI9gBaebbnrfifHhDYfgasaacH8akY=wiFfYdH8Gipec8Eeeu0xXdbba9frFj0=OqFfea0dXdd9vqai=hGuQ8kuc9pgc9s8qqaq=dirpe0xb9q8qiLsFr0=vr0=vr0dc8meaabaqaciaacaGaaeqabaqabeGadaaakeaacqWGnbqtcqWGZbWCcqGH9aqpcqWGnbqtdaWgaaWcbaGaem4CamNaem4Ba8gabeaakiabgwSixlabdwgaLnaaCaaaleqabaGaeyOeI0IaeiikaGIaem4AaS2aaSbaaWqaaiabd2gaTbqabaWccqGHRaWkcqWGYbGCdaWgaaadbaGaemiCaahabeaaliabcMcaPiabgwSixlabdsha0baaaaa@4581@

where M_S _(μg) is the mass of TBA (M_S _= C·V_S_, where V_S _is the volume of the slurry in liters) in the slurry at time t, M_SO _is the initial mass of TBA at t = 0, and t is time in days. Additionally, the rate of change of TBA that included mineralization and removal via partitioning can also be determined with equations 3 and 4

dMmdt=km⋅Ms
 MathType@MTEF@5@5@+=feaafiart1ev1aaatCvAUfKttLearuWrP9MDH5MBPbIqV92AaeXatLxBI9gBaebbnrfifHhDYfgasaacH8akY=wiFfYdH8Gipec8Eeeu0xXdbba9frFj0=OqFfea0dXdd9vqai=hGuQ8kuc9pgc9s8qqaq=dirpe0xb9q8qiLsFr0=vr0=vr0dc8meaabaqaciaacaGaaeqabaqabeGadaaakeaadaWcaaqaaiabdsgaKjabd2eannaaBaaaleaacqWGTbqBaeqaaaGcbaGaemizaqMaemiDaqhaaiabg2da9iabdUgaRnaaBaaaleaacqWGTbqBaeqaaOGaeyyXICTaemyta00aaSbaaSqaaiabdohaZbqabaaaaa@3C91@

dMpdt=rp⋅Ms
 MathType@MTEF@5@5@+=feaafiart1ev1aaatCvAUfKttLearuWrP9MDH5MBPbIqV92AaeXatLxBI9gBaebbnrfifHhDYfgasaacH8akY=wiFfYdH8Gipec8Eeeu0xXdbba9frFj0=OqFfea0dXdd9vqai=hGuQ8kuc9pgc9s8qqaq=dirpe0xb9q8qiLsFr0=vr0=vr0dc8meaabaqaciaacaGaaeqabaqabeGadaaakeaadaWcaaqaaiabdsgaKjabd2eanjabdchaWbqaaiabdsgaKjabdsha0baacqGH9aqpcqWGYbGCdaWgaaWcbaGaemiCaahabeaakiabgwSixlabd2eanjabdohaZbaa@3C49@

where M_m _(μg) and M_p _(μg) are the mass of TBA mineralized and partitioned, respectively. Substitution of equation 2 into equations 3 and 4 and integration of each equation provides

Mm=kmMsokm+rp(1−e−(km+rp)⋅t)
 MathType@MTEF@5@5@+=feaafiart1ev1aaatCvAUfKttLearuWrP9MDH5MBPbIqV92AaeXatLxBI9gBaebbnrfifHhDYfgasaacH8akY=wiFfYdH8Gipec8Eeeu0xXdbba9frFj0=OqFfea0dXdd9vqai=hGuQ8kuc9pgc9s8qqaq=dirpe0xb9q8qiLsFr0=vr0=vr0dc8meaabaqaciaacaGaaeqabaqabeGadaaakeaacqWGnbqtdaWgaaWcbaGaemyBa0gabeaakiabg2da9maalaaabaGaem4AaS2aaSbaaSqaaiabd2gaTbqabaGccqWGnbqtdaWgaaWcbaGaem4CamNaem4Ba8gabeaaaOqaaiabdUgaRnaaBaaaleaacqWGTbqBaeqaaOGaey4kaSIaemOCai3aaSbaaSqaaiabdchaWbqabaaaaOWaaeWaaeaacqaIXaqmcqGHsislcqWGLbqzdaahaaWcbeqaaiabgkHiTmaabmaabaGaem4AaS2aaSbaaWqaaiabd2gaTbqabaWccqGHRaWkcqWGYbGCdaWgaaadbaGaemiCaahabeaaaSGaayjkaiaawMcaaiabgwSixlabdsha0baaaOGaayjkaiaawMcaaaaa@5096@

Mp=rpMsokm+rp(1−e−(km+rp)⋅t)
 MathType@MTEF@5@5@+=feaafiart1ev1aaatCvAUfKttLearuWrP9MDH5MBPbIqV92AaeXatLxBI9gBaebbnrfifHhDYfgasaacH8akY=wiFfYdH8Gipec8Eeeu0xXdbba9frFj0=OqFfea0dXdd9vqai=hGuQ8kuc9pgc9s8qqaq=dirpe0xb9q8qiLsFr0=vr0=vr0dc8meaabaqaciaacaGaaeqabaqabeGadaaakeaacqWGnbqtdaWgaaWcbaGaemiCaahabeaakiabg2da9maalaaabaGaemOCai3aaSbaaSqaaiabdchaWbqabaGccqWGnbqtdaWgaaWcbaGaem4CamNaem4Ba8gabeaaaOqaaiabdUgaRnaaBaaaleaacqWGTbqBaeqaaOGaey4kaSIaemOCai3aaSbaaSqaaiabdchaWbqabaaaaOWaaeWaaeaacqaIXaqmcqGHsislcqWGLbqzdaahaaWcbeqaaiabgkHiTmaabmaabaGaem4AaS2aaSbaaWqaaiabd2gaTbqabaWccqGHRaWkcqWGYbGCdaWgaaadbaGaemiCaahabeaaaSGaayjkaiaawMcaaiabgwSixlabdsha0baaaOGaayjkaiaawMcaaaaa@50B0@

Since the mass of TBA partitioned was not measured in this study, the expected mass [M_pm _(μg)], was determined through a mass balance equation based on the measured mineralization of TBA [M_mm _(μg)] and the predicted mass remaining in the slurry (M_S_).

*M*_*pm *_= *M*_*so *_- (*M*_*s *_+ *M*_*mm*_)

k_m _and r_p _values were determined through minimizing the sum of the differences squared of the measured or expected values (M_mm _and M_pm_) and the predicted values (M_m _and M_p_). This approach assumes that all samples have the same variance.

min⁡=∑((Mmm−Mm)Mm)2+∑((Mpm−Mp)Mp)2
 MathType@MTEF@5@5@+=feaafiart1ev1aaatCvAUfKttLearuWrP9MDH5MBPbIqV92AaeXatLxBI9gBaebbnrfifHhDYfgasaacH8akY=wiFfYdH8Gipec8Eeeu0xXdbba9frFj0=OqFfea0dXdd9vqai=hGuQ8kuc9pgc9s8qqaq=dirpe0xb9q8qiLsFr0=vr0=vr0dc8meaabaqaciaacaGaaeqabaqabeGadaaakeaacyGGTbqBcqGGPbqAcqGGUbGBcqGH9aqpdaaeabqaamaabmaabaWaaSaaaeaadaqadaqaaiabd2eannaaBaaaleaacqWGTbqBcqWGTbqBaeqaaOGaeyOeI0Iaemyta00aaSbaaSqaaiabd2gaTbqabaaakiaawIcacaGLPaaaaeaacqWGnbqtdaWgaaWcbaGaemyBa0gabeaaaaaakiaawIcacaGLPaaaaSqabeqaniabggHiLdGcdaahaaWcbeqaaiabikdaYaaakiabgUcaRmaaqaeabaWaaeWaaeaadaWcaaqaamaabmaabaGaemyta00aaSbaaSqaaiabdchaWjabd2gaTbqabaGccqGHsislcqWGnbqtdaWgaaWcbaGaemiCaahabeaaaOGaayjkaiaawMcaaaqaaiabd2eannaaBaaaleaacqWGWbaCaeqaaaaaaOGaayjkaiaawMcaaaWcbeqab0GaeyyeIuoakmaaCaaaleqabaGaeGOmaidaaaaa@5498@

Rate constants at the three specified temperatures were then applied to the Arrhenius equation for development of the Arrhenius constants: frequency factor (A, day^-1^) and activation energy (E_a_, mol/J) as seen in the equation

kT=A⋅exp⁡(EaR⋅T)
 MathType@MTEF@5@5@+=feaafiart1ev1aaatCvAUfKttLearuWrP9MDH5MBPbIqV92AaeXatLxBI9gBaebbnrfifHhDYfgasaacH8akY=wiFfYdH8Gipec8Eeeu0xXdbba9frFj0=OqFfea0dXdd9vqai=hGuQ8kuc9pgc9s8qqaq=dirpe0xb9q8qiLsFr0=vr0=vr0dc8meaabaqaciaacaGaaeqabaqabeGadaaakeaacqWGRbWAdaWgaaWcbaGaemivaqfabeaakiabg2da9iabdgeabjabgwSixlGbcwgaLjabcIha4jabcchaWnaabmaabaWaaSaaaeaacqWGfbqrdaWgaaWcbaGaemyyaegabeaaaOqaaiabdkfasjabgwSixlabdsfaubaaaiaawIcacaGLPaaaaaa@40D6@

where k_T _is a first order rate (day^-1^) at temperature T (K) and R is the ideal gas constant (8.314 J/mol·K). Equation 9 can be linearized by taking the natural log of both sides, that yields:

ln⁡(kT)=−EaR⋅T+ln⁡(A)
 MathType@MTEF@5@5@+=feaafiart1ev1aaatCvAUfKttLearuWrP9MDH5MBPbIqV92AaeXatLxBI9gBaebbnrfifHhDYfgasaacH8akY=wiFfYdH8Gipec8Eeeu0xXdbba9frFj0=OqFfea0dXdd9vqai=hGuQ8kuc9pgc9s8qqaq=dirpe0xb9q8qiLsFr0=vr0=vr0dc8meaabaqaciaacaGaaeqabaqabeGadaaakeaacyGGSbaBcqGGUbGBdaqadaqaaiabdUgaRnaaBaaaleaacqWGubavaeqaaaGccaGLOaGaayzkaaGaeyypa0ZaaSaaaeaacqGHsislcqWGfbqrdaWgaaWcbaGaemyyaegabeaaaOqaaiabdkfasjabgwSixlabdsfaubaacqGHRaWkcyGGSbaBcqGGUbGBdaqadaqaaiabdgeabbGaayjkaiaawMcaaaaa@433C@

Plotting the ln(k_T_) vs 1/RT will provide a line with intercept of ln(A) and slope of E_a_.

### Mineralization rates

**Figure 3:**
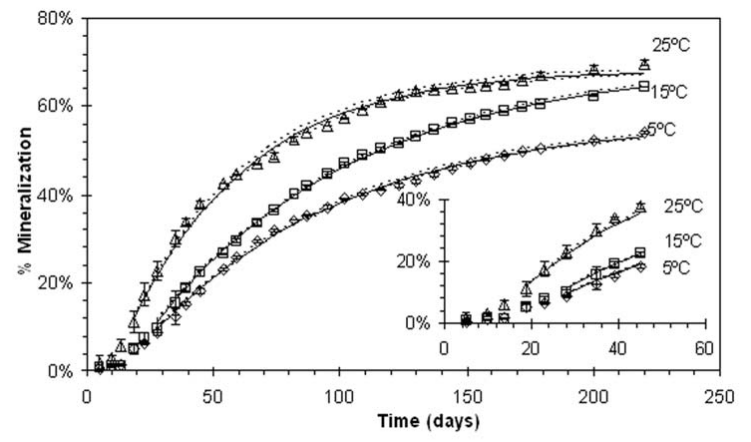
**Cumulative measured TBA mineralization showing lag phase and model fit**. Non-poison microcosms at (◇)5°C, (□)15°C, and (△)25°C. Error bars represent standard deviation. Solid and dashed lines represent the predicted mass mineralized (M_m_) and 95% upper and lower confidence limits. Model does not include the lag phase (days 0–23 for the 5°C and 15°C microcosms and days 0–14 for the 25°C microcosms).

Mineralization of TBA at 5°C and 15°C each had lag phases of 23 days before entering a log phase of mineralization, and microcosms incubated at 25°C had a lag phase of 14 days (Figure 3). Regression, using equation 5 and 6, and data plots with 95% upper and lower confidence intervals for increase in mineralization with time are presented in Figure 3. Reports presented in Table 1 include first order kinetic rate constants for mineralization (k_m_) and removal rate constants via partitioning (r_p_) of TBA determined from equations 5 and 6, total percent mineralization, initial concentration at the end of the lag phase (M_SO_), and the minimum of the sum of the differences squared for each incubation temperature. Mineralization rates of TBA at the three temperatures where shown to be statistically different by using the method of least squares.

**Table 1:** Mineralization results. k_m_, r_v_, total percent mineralization, M_SO_, the minimum of the sum of the least squares values for microcosms at 5°C, 15°C, and 25°C

	Temperature (°C)		
	
	5	15	25
	7.84 ± 0.14 × 10^-3^	9.07 ± 0.09 × 10^-3^	15.3 ± 0.3 × 10^-3^
k_m _(days^-1^)			
k_m _(years^-1^)	2.86 ± 0.05	3.31 ± 0.03	5.60 ± 0.14
r_p _(days^-1^)	4.89 × 10^-3^	5.36 × 10^-3^	5.88 × 10^-3^
Total mineralization	54.1%	64.4%	69.8%
M_SO _(mg/L)	1.70	1.66	1.73
Sum Δ^2^	0.16	1.50	0.50

M_SO_: initial mass of TBA in the slurry after the lag phase, k_m_: first order biodegradation kinetic rate constant, r_p_: average rate of partitioning of TBA from the slurry

Comparison of TBA mineralization rates observed in this study to other published values can be seen in Table 2. Only two studies have reported degradation rates for TBA and both were under different redox conditions [19,20]. The study by Schirmer et al. [19] used microcosms with soil from the aquifer at the Canadian Force Base (CFB) Borden (Ontario, Canada). The Borden aquifer was used in a natural gradient experiment with dissolved MTBE-containing gasoline. The experiment started in 1988 and was originally designed to observe the transport of MTBE in the subsurface. In 1996 MTBE was observed to be present at only 3% of the original mass (taking into account dispersion and dilution). The aquifer is underlined by an impermeable clayey layer that sits on top of a confined aquifer with no detectable measurements of MTBE in the confined layer. MTBE was assumed to be removed via biodegradation but the presence of daughter compounds, TBA and TBF, where not detected. Schirmer et al. [19] used microcosms with soils from the Borden aquifer to determine the potential of MTBE and TBA biodegradation. MTBE and TBA were found to degrade with rate constants of 0.04–0.07 day^-1 ^and 0.12 day^-1^, respectively, at 10°C. The authors postulated that TBA did not accumulate because its biodegradation rate was significantly greater than MTBE.

**Table 2:** TBA degradation study comparison

Terminal Electron Acceptor	TBA Degradation	Degradation Rate	Temp. (°C)	Reference
Oxygen	54.0% over 220 days	7.84 × 10^-3 ^d^-1^	5	This Study
	64.4% over 220 days	9.07 × 10^-3 ^d^-1^	15	This Study
	69.8% over 220 days	15.3 × 10^-3 ^d^-1^	25	This Study
	99% over 198 days	ni	nr	[17]
	70% over 105 days	ni	nr	[18]
	TBA accumulation	0.12 d^-1^	10	[19]
Nitrate	49% over 198 days	ni	nr	[17]
Sulfate	5% over 198 days	ni	nr	[17]
	*in situ*	0.26 – 1.1 year^-1^	16-7	[20]
Fe (III)	25% over 65 days	ni	nr	[21]
Mn(IV)	75% over 198 days	ni	nr	[17]

ni: not investigated, nr: not reported

The removal of TBA at the Ronan site at 25°C is significantly less than at the Borden site at 10°C (0.0153 day^-1 ^compared to 0.12 day^-1^) [19]. However, this is not unexpected since the Ronan site has a measurable presence of TBA (up to 1000 μg/L) [16].

The study by Day and Gulliver [20] was performed in situ using TBA carbon isotopes (^13^C) to follow the fate and transport of TBA in a contaminated shallow aquifer. This is a unique study because TBA was introduced into the aquifer by a surface release of TBA at a chemical plant in Pasadena, Texas and was not introduced via MTBE degradation. The aquifer was determined to be under sulfate reducing conditions and researchers observed TBA first order degradation rate constants of 0.26, 0.97, and 1.1 year^-1 ^with temperature range of 7–16°C. The rate constants observed by Day and Guliver [20] are significantly slower than constants observed in this study (ranging from 2.86 to 5.60 years^-1^), which were determined under oxic conditions. This is not unexpected since TBA and MTBE degradation has been observed to be slower under sulfate reducing conditions [17-19,21].

### Temperature effects

The effect of temperature on TBA biodegradation in this study was statistically significant. Application of the data to the linear Arrhenius equation (equation 10) yields constant values of constants A and Ea of 154 day^-1 ^and 23,006 mol/J, respectively. Although no other published study has reported a TBA degradation kinetic experiment at different temperatures, a study was performed with MTBE on a Ronan sediment at four temperatures (4°C, 14°C, 24°C, and 34°C) by Bradley and Landmeyer [5]. The authors observed that MTBE was mineralized at the fastest rate at 24°C and decreased with temperature down to 4°C (incubated for 77 days).

**Figure 4:**
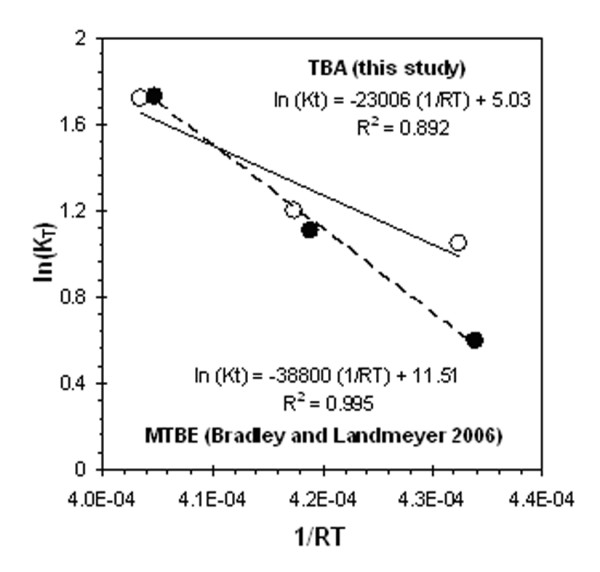
**Temperature effects on MTBE and TBA biodegradation rates**. Rates from this study for TBA (○) and Bradley and Landmeyer [5] for MTBE (●) applied to the Arrhenius equation.

Calculation of first order rate constants for MTBE from the data presented in Bradley and Landmeyer [5] yield 4.95 × 10^-3^, 8.30 × 10^-3^, 15.4 × 10^-3 ^and 7.09 × 10^-3 ^day-1 values for 4°C, 14°C, 24°C, and 34°C microcosms, respectively. These rates applied to the Arrhenius equation are comparable to the rates determined for TBA in this study (Figure 4) and yield values for A and Ea of 99,509 day^-1 ^and 38,800 mol J^-1^. The kinetic rate constant determined for MTBE degradation at 34°C was not included in the regression since the Arrhenius equation only models continuous increase in kinetic rates with increases in temperature; also, the annual groundwater temperature at the Ronan site ranges from 14°C to 5°C and does not exceed 24°C.

Placing the A and Ea values into equation 9 for MTBE and TBA, setting the equations equal to each other, and solving for T provides the temperature where MTBE and TBA degradation rates are equal, which is 20.4°C. Therefore, temperatures below 20.4°C will induce kinetic rate constants that are faster for TBA than MTBE. Since the temperature at the Ronan site is below 20.4°C, it could be assumed that aerobic TBA biodegradation is faster than MTBE. This theory agrees with findings in other published studies that observed similar rates [17,22,23].

The presence of TBA at the Ronan site could be explained by redox conditions in the subsurface in the hyporheic zone. Multiple studies have found that TBA accumulates under anaerobic conditions and most subsurface environments, especially in hyporheic soils and sediments, do not have homogeneous redox conditions, but include both anoxic and oxic regions [17,21,24].

## Discussion and conclusion

Results from this study have confirmed that TBA degradation is occurring at the Ronan, MT site and that the rate of removal of TBA is affected by temperature. These results provide support of the application of MNA at the site, since it has been demonstrated in laboratory experiments that indigenous microorganisms at the site are capable of degrading both MTBE and TBA.

Biodegradation of TBA should be evaluated further by investigating the effects of dissolved oxygen (DO) concentrations on TBA mineralization rate. Degradation studies with polycyclic aromatic hydrocarbons (PAHs) and pentachlorophenol (PCP) showed that ambient oxygen concentrations in the soil gas phase could be reduced to 2% v/v (or approximately 0.8 mg/L in the soil aqueous phase) with no significant reduction in mineralization kinetics [25,26]. Reporting the effect of DO concentrations in the gaseous phase of the Ronan, MT hyporheic zone soils on the rate and extent of TBA mineralization is planned.

This study also has implications in developing microbiology tools to help determine the use of MNA as remediation. Determination of microbial strains responsible for TBA and MTBE biodegradation at the Ronan site could be used to create a genetic primer to identify other viable microbes at MTBE and TBA contaminated sites. The use of this primer would assist in understanding the ability of the site to degrade the constituents.

## Abbreviations

**A**: frequency factor; **BaCl**: barium chloride; **bgs**: below ground surface; **C**: concentration of TBA; **CO**_2_: carbon dioxide; **DOC**: dissolved organic carbon; **DO**: dissolved oxygen; **E**_a_: activation energy; **ETBE**: ethyl tert-butyl ether; **HgCl**_2_: mercuric chloride; **k**_m_: first order mineralization rate; **KOH**: potassium hydroxide; **LUFT**: leaking underground fuel tank; **M**_m_: measured mass of TBA mineralized; **M**_mm_: modeled mass of TBA mineralized; **MNA**: monitored natural attenuation; **M**_p_: mass of TBA partitioned; **M**_pm_: modeled mass of TBA partitioned; **M**_S_: mass of TBA in slurry; **M**_SO_: initial mass of TBA in slurry; **MTBE**: methyl tert-butyl ether; **PAH**: polycyclic aromatic hydrocarbon; **PCP**: pentachlorophenol; **R**: ideal gas constant; **r**_p_: first order partitioning rate; **T**: temperature; **t**: time; **TBA**: tert-butyl alcohol; **TBF**: ter-butyl formate; **VOA**: volatile organic air-tight; **V**_S_: volume of slurry

## Competing interests

The author(s) declare that they have no competing interests.

## Authors' contributions

MHG carried out field sampling, mineralization studies, derived the mineralization model and drafted the manuscript. RCS conceived the study and participated in its design. JEM participated in data interpretation and statistical analysis and WJD assisted in microcosm design and empirical modeling of the data. All authors read and approved the final manuscript.
